# Attention impairment in patients with cervical dystonia: An attention network test study

**DOI:** 10.3389/fpsyg.2022.952567

**Published:** 2022-08-04

**Authors:** Kun Xia, Yongsheng Han, Lanlan Zhou, Sheng Hu, Rao Rao, Shu Shan, Lei Hua

**Affiliations:** ^1^Institute of Neurology, Anhui University of Chinese Medicine, Hefei, China; ^2^Department of Neurology, Anhui Hospital of Integrated Traditional Chinese and Western Medicine, Hefei, China; ^3^Department of Neurology, The First Affiliated Hospital of Anhui University of Traditional Chinese Medicine, Hefei, China; ^4^School of Medical Information Engineering, Anhui University of Traditional Chinese Medicine, Hefei, China

**Keywords:** cervical dystonia, cognitive, attention, attention network test, alerting

## Abstract

**Objective:**

The purpose of this study was to investigate attentional network functional characteristics in patients with cervical dystonia (CD).

**Methods:**

A total of 29 patients with CD and 26 healthy controls (HCs) were recruited. All subjects participated in the study and underwent the Attention Network Test (ANT), which evaluated the efficiencies of three independent attention networks (alerting, orienting, and executive control), as well as reaction time (RT) and accuracy.

**Results:**

Significant differences between CD patients (9.86 ± 27.95 ms) and HCs (33.62 ± 23.41 ms) were observed in the alerting network (*t* = −3.40, *p* < 0.05). In contrast, the orienting network (*t* = 0.26, *p* = 0.79), executive control network (*Z* = −0.55, *p* = 0.58), total mean reaction time (*t* = −2.6, *p* = 0.79), and total accuracy rate (*Z* = −1.67, *p* = 0.09) showed no significant differences between the two groups.

**Conclusion:**

Patients with CD showed a significant deficit in the alerting network. However, they did not show any deficits in the orienting or executive control network. In addition, the alerting, orienting, and executive control network functions of CD patients were all affected by the severity of torticollis, especially the alerting network function.

## Introduction

Cervical dystonia (CD) is the most common type of focal dystonia in adults. It is characterized by continuous or intermittent involuntary contraction of cervical muscles, leading to involuntary torsion, lateral tilt, flexion, and backwardness of the head and neck (Stacy, 2008). In addition to symptoms of movement disorders, patients with CD may also experience nonmotor symptoms such as pain, anxiety, depression, sleep disorders, sexual dysfunction, and cognitive dysfunction (Charles et al., 2016; Hertenstein et al., 2016; Yang et al., 2017; Marek et al., 2018). In the present neuropsychological studies of CD, the study of cognitive function is the mainstream research direction. Relevant studies have shown that the overall cognitive performance of CD patients is normal, as measured by the Mini Mental State Examination (MMSE), but exhibits significant defects in assessments related to specific cognitive skills, such as social cognitive function, executive function, and attention function (Romano et al., 2014; Czekóová et al., 2017; Bastos et al., 2021; Monaghan et al., 2021). Attention is an important aspect of cognitive function. However, only a few studies have focused on attention function in patients with CD. Chillemi et al. (2018) found that the visual spatial attention of CD patients is abnormal. Scott et al. (2003) found that patients with primary dystonia have defects in attention and executive function. Duane (2004) stated that the deficits in executive function and attention of CD patients are the most prominent, and these deficits may not be secondary to motor symptoms. Although some studies have revealed that CD patients have attention dysfunction, the current research on the attention function of CD patients is not thorough or comprehensive. Attention is a prerequisite for all high-level cognitive processes, and the understanding of attention has reached a deeper level based on a large amount of neuroanatomical and cognitive research. Posner and Petersen (1990) divided attention into three independent subnetworks: alerting, orienting, and executive control, and each of them corresponds to a specific anatomical region and neurotransmitter. The Attentional Network Test (ANT; Fan et al., 2002) was designed according to Posner’s attentional theory (Posner and Petersen, 1990). The ANT can not only perform a fine quantitative analysis of the subjects’ attention network function but also may have auxiliary diagnostic value as a basis for neuropsychological evaluation. Currently, there are no reports on the attention network function of CD patients. Whether CD patients have comprehensive attention deficits or specific attention network deficits are currently unclear. To clarify the characteristics of the impaired attention network function of patients with CD, this study was carried out as detailed below.

## Materials and methods

### Participants

Twenty-nine patients with CD were recruited from the Institute of Neurology, Anhui University of Traditional Chinese Medicine, China. The CD subtype distribution according to the Col-Cap concept (Jost and Tatu, 2015) was as follows: 18 patients with torticollis type, 4 patients with laterocollis type, 2 patients with retrocaput type, 2 patients with antecaput type, 2 patients with laterocaput type, and 1 patient with retrocollis type. The inclusion criteria were as follows: (1) idiopathic and focal CD (Albanese et al., 2013); (2) not receiving relevant drugs, surgical treatment, or botulinum toxin treatment for the first time or in the past 3 months; (3) right-handed; and (4) no organic changes found on MRI scan. The exclusion criteria included the following: (1) secondary torticollis caused by trauma, drugs, intracranial lesions, or neurodegenerative diseases; (2) patients with intellectual disability or hearing, visual or language impairment; (3) patients with mental disorders; (4) patients taking central anticholinergic drugs, antipsychotics, or other drugs that can affect attention; (5) patients with apparent tremor of the head and abnormal posture of the neck that cannot be corrected by external forces; and (6) those who could not complete the experiment.

Twenty-six individuals matched for sex, age, and education level of CD patients were selected from the community to form a healthy control (HC) group. All the members of the HC group were right-handed, without visual acuity, hearing, speaking, writing, or understanding impairment. There were no central nervous system diseases, no systemic chronic diseases that may lead to mental symptoms or cognitive dysfunction, and no drugs that may affect attention.

This study was conducted in accordance with the Declaration of Helsinki. The research protocol was approved by the ethics committee of the Affiliated Hospital of Institute of Neurology of Anhui University of Traditional Chinese Medicine. All participants provided written informed consent.

### Demographic data

The clinical information for all subjects was recorded in the form of questionnaires, including sex, age, education level, current medical history, past medical history, family history, medication history, and sensory tricks. In addition, the CD group underwent assessment using the Toronto Western Spasmodic Torticollis Rating Scale (TWSTRS; Espay et al., 2018) to evaluate the severity of symptoms.

### Neuropsychological background tests

All subjects underwent the same neuropsychological scale assessment, and the results were further compared between the two groups. The neuropsychological tests applied were as follows: (1) the Montreal Cognitive Assessment Beijing Version (MoCA-BJ) was used to evaluate intelligence (Tian et al., 2020); (2) the Hamilton Anxiety Scale (HAMA; Gjerris et al., 1983) was used to measure anxiety states; and (3) the Hamilton Depression Scale (HAMD; Miller et al., 1985) was used to assess depressive states.

### Attention network test

**Figure 1:**
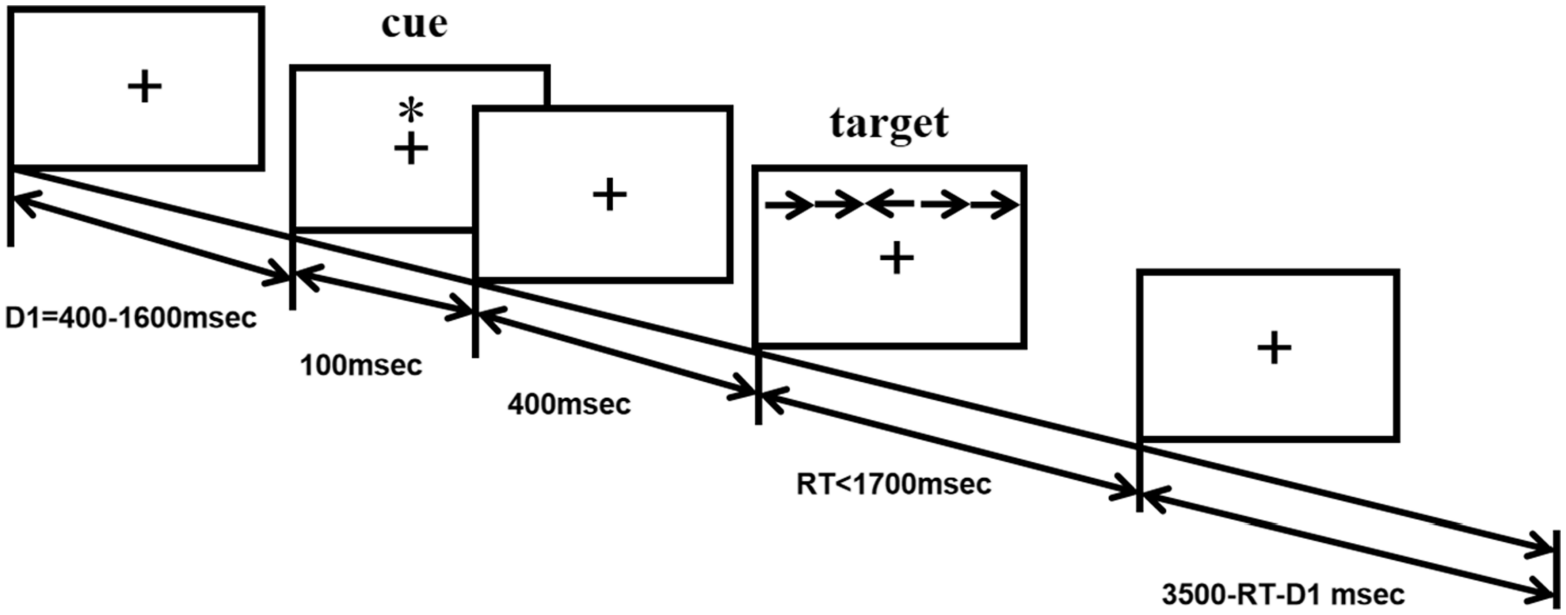
Experimental paradigm.

**Figure 2:**
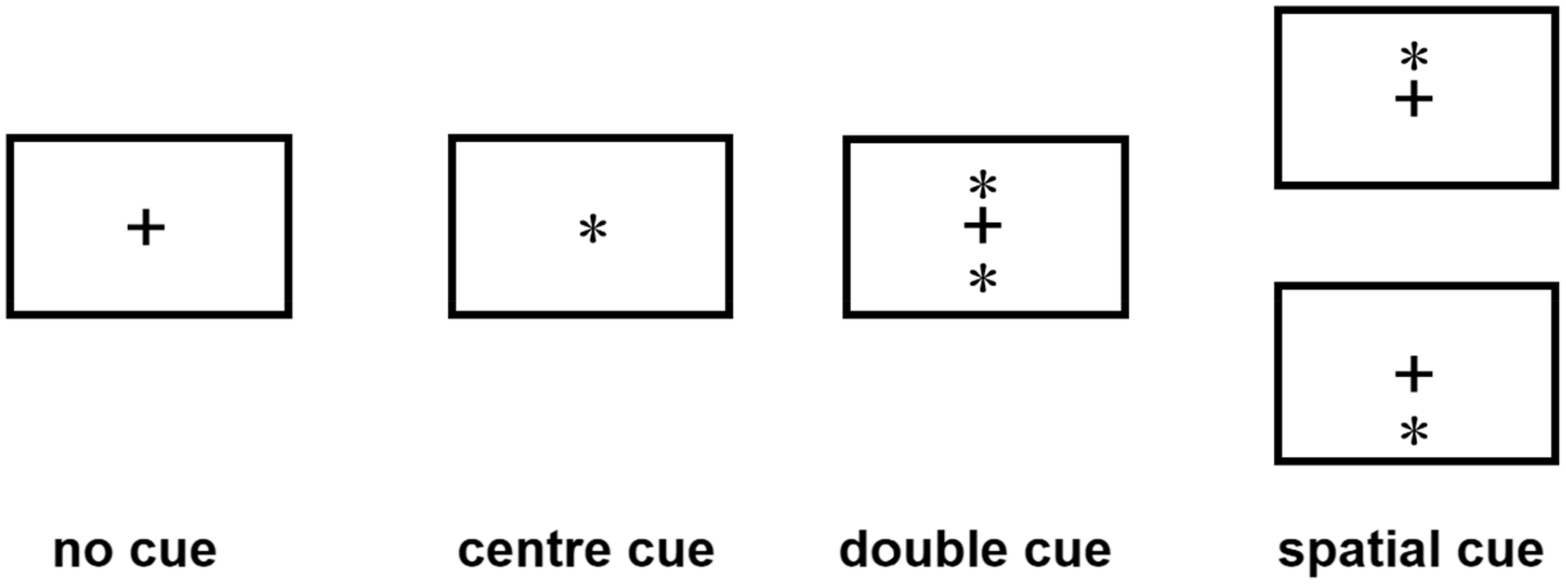
Cue conditions.

**Figure 3:**
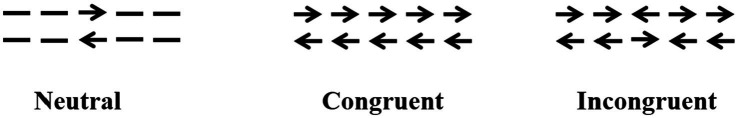
Target conditions.

The ANT program (Fan et al., 2002) was programmed using E-Prime software and runs on a14.1-inch laptop. Each test procedure includes five steps (see Figure 1). First, the fixation point “+” (400–1,600 ms, D1) is presented in the center of the screen; second, the cue condition is presented (100 ms); third, the fixation point “+” in the center is presented (400 ms); and fourth, the target stimulus is presented (400 ms). The target stimulation appears in the spatial position above or below the fixation point “+.” The patient needs to respond to the direction of the target stimulation arrow as soon as possible. When the patient presses the key, the target stimulation disappears immediately, and this period of time does not exceed 2,700 ms. Fifth, the gaze point appears in the center of the screen. The total time of each test procedure was 5,000 ms. There were four types of cue conditions for the second step of the test: (1) no cue, “*” appeared; (2) a central cue, in the position where “*” appeared coincided with “+”; (3) a double cue, “*” appeared above and below “+” at the same time; and (4) a spatial cue, “*,” was displayed above or below “+,” which was the same as the target stimulus position that subsequently appeared. The spatial cue belongs to the effective space suggestion, while the center cue and the double cue belong to the invalid space suggestion, as shown in Figure 2. There are three types of target stimuli for the third step of the test: (1) neutral: a leftward or rightward pointing arrowhead flanked on either side by lines without arrowheads; (2) congruent flankers: the direction of the 5 arrows is the same; and (3) incongruent flankers: the directions of the two arrows on the left and right are not consistent with the direction of the middle arrow (see Figure 3 for details). Participants were required to determine the direction of the central arrow and press either the left key or right key accordingly. The computer automatically recorded the participant’s reaction time (RT) and accuracy. Before the test, the established instructions were used to familiarize the participants with the requirements of the test. At the beginning of the test, 24 pretests were conducted to familiarize the participants with the test operation in advance. The formal test was conducted 312 times, and the participants were divided into three groups. After the test for each group was completed, the subjects could rest for 5 min in the middle, and the total time was approximately 30 min. During the examination, the participants were required to keep their heads neutral and face the computer screen at a distance of approximately 40 cm. Patients with CD could support their head and face with their palms or lean their heads against a wall, keeping their heads as neutral as possible.

### Calculation of attention network efficiencies

The efficiency of the alerting, orienting, and executive control networks can be calculated by the principle of subtraction of RTs under different conditions (Fan et al., 2002). The calculation equation is as follows: alerting network efficiency was measured as RTs with no cue conditions minus RTs with double cue conditions, where a larger value indicates a higher efficiency of the alerting network; orienting network efficiency was calculated as RTs with a center cue minus RTs with a spatial cue, where a larger value indicates a directional network with higher efficiency; and executive control network efficiency was equal to RTs of the incongruent flankers minus RTs in the conditions with the congruent flankers, where a smaller value indicates a higher efficiency of the executive function network.

### Statistical analysis

SPSS 26.0 software was used for data analysis. All continuous measurements are presented as the mean ± SD. Two independent-sample *t*-tests were used to compare two groups of measurement data conforming to a normal distribution; the Mann–Whitney *U* test was performed on continuous variables with a nonnormal distribution; and the χ^2^ test was used to compare count data. A 2 (group: CD, HC) × 4 (cue type: no cue, double cue, central cue, and spatial cue) × 3 (target type: congruent, incongruent, and neutral) mixed factor ANOVA on RT was carried out. Spearman correlations were used to determine the relationship between the patients’ performance on the ANT and their clinical or neuropsychological ability. For the two-tailed test, the significance level was set to *p* < 0.05.

## Results

### Demographic and clinical data

There were no significant differences between the two groups with respect to demographic data, including sex, age, and education. The neuropsychological data of MoCA-BJ, HAMA, and HAMD scores were also not significantly different between the two groups, as shown in Table 1.

**Table 1:** Demographic data and neuropsychological background data of the CD and HC groups.

	CD (*N* = 29)	HC (*N* = 26)	*P*
Sex (M/F)	12/17	13/13	0.522
Age at investigation (years, mean ± SD)^a^	39.83 (11.34)	39.46 (10.75)	0.903
Years of school education (years, mean ± SD)^a^	10.45 (3.61)	10.23 (4.02)	0.833
TWSTRS score (mean ± SD)^a^	32.62 (4.75)		
Severity Scale score (mean ± SD)^a^	19.66 (2.53)		
Disability Scale score (mean ± SD)^a^	11.24 (1.81)		
Pain Scale score (mean ± SD)^a^	1.66 (1.14)		
MoCA-BJ score (mean ± SD)^b^	26.28 (2.99)	26.12 (3.77)	0.861
HAMA (mean ± SD)^b^	6.00 (3.57)	4.96 (3.70)	0.187
HAMD (mean ± SD)^b^	5.52 (4.14)	4.46 (3.13)	0.532

a Normally distributed, intergroup comparison using the independent-sample *t*-test.

b Nonnormally distributed data, intergroup comparison using the Mann–Whitney U test.

### The efficiencies of the three networks

**Figure 4:**
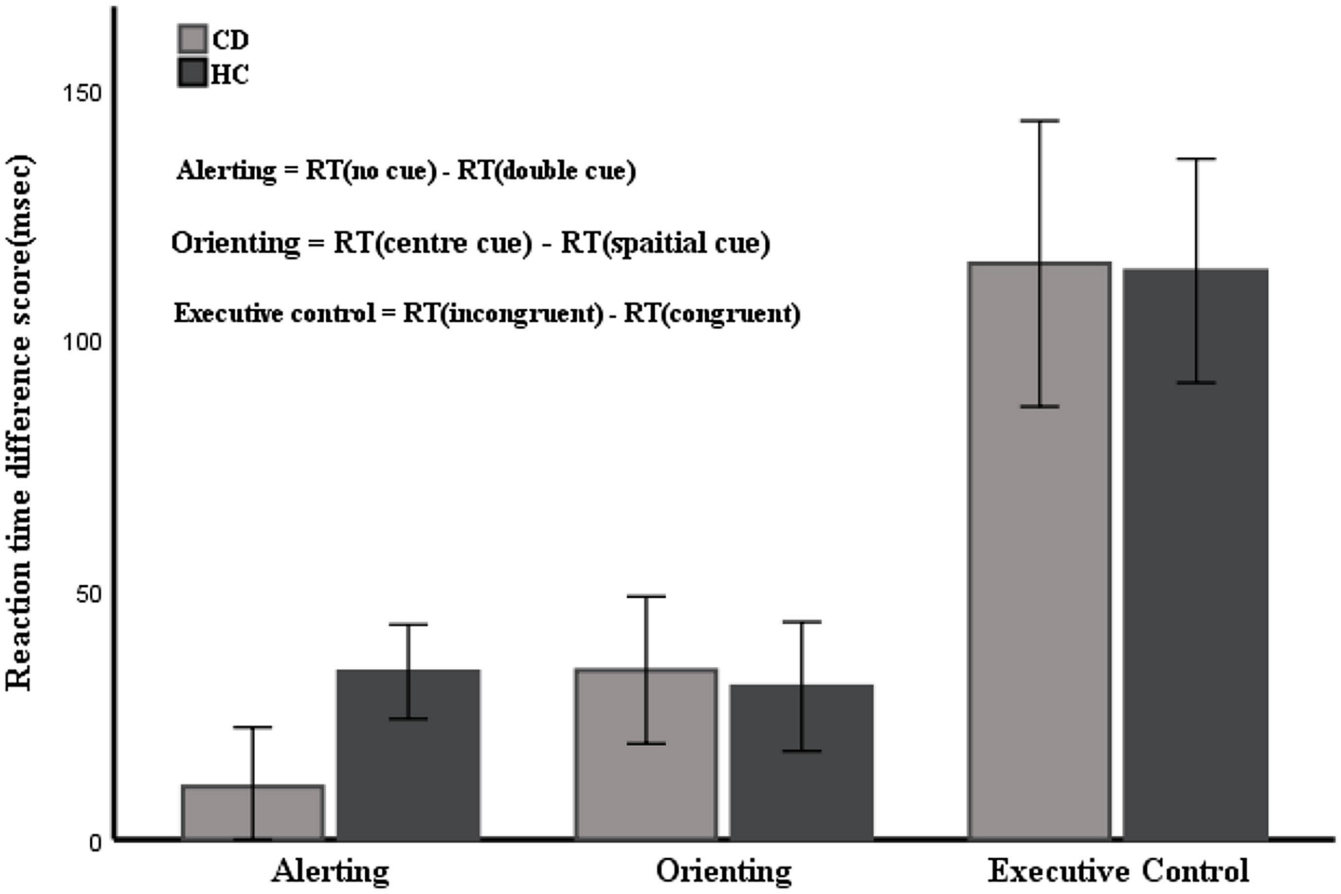
Mean RTs of the alerting, orienting, and executive network. Error bars represent mean standard errors.

Table 2 shows the mean RTs under each condition of the two groups. The main effect of group was not significant [*F*(1, 53) = 0.122, *p* = 0.728]. The main effects of cue type and target type were significant [*F*(3, 159) = 22.369, *F*(2, 106) = 300.128, respectively; *p* < 0.01]. There were significant interactions between cue type and target type [*F*(6, 318) = 2.518, *p* = 0.021]. No significant interactions were found between group and target type [F(2, 106) = 0.079, *p* = 0.924] or group and cue type [F(3, 159) = 0.542, *p* = 0.654]. The three-way interaction between group, cue type, and target type was also not significant [F(6, 318) = 0.586, *p* = 0.742]. Table 3 and Figure 4 show the mean score and the standard error (SE) for each of the attention networks, the total mean RT, and the global accuracy. The efficiency of the alerting network in the CD group was significantly lower than that in the HC group (*t* = −3.40, *p* = 0.01); the efficiency of the orienting network in the CD group was slightly higher than that of the HC group, but the difference was not statistically significant (*t* = 0.26, *p* = 0.79); the efficiency of the executive control network in the CD group was slightly lower than that of the HC group, but the difference was not statistically significant (*t* = −0.55, *p* = 0.58). The total mean RT and the accuracy rates were similar in the two groups (*t* = −0.26, *p* = 0.79; *Z* = −1.67, *p* = 0.09).

**Table 2:** Mean RTs under each condition in patients with CD and HCs.

Group		Target type	Cue type			
			No cue	Double cue	Center cue	Spatial cue
**Mean RTs (ms) and standard deviations**						
CD		Congruent	718.14 (111.25)	696.59 (148.73)	707.14 (134.75)	658.38 (164.07)
		Incongruent	814.10 (85.76)	795.66 (117.20)	823.52 (93.78)	765.66 (121.91)
		Neutral	623.34 (115.26)	601.59 (112.62)	611.07 (123.29)	593.31 (129.59)
HCs		Congruent	734.04 (104.15)	692.85 (115.63)	704.85 (107.86)	677.62 (109.01)
		Incongruent	824.27 (92.36)	808.08 (89.01)	823.35 (91.15)	776.88 (93.47)
		Neutral	648.73 (102.11)	611.69 (102.13)	621.15 (102.74)	600.15 (111.41)

**Table 3:** Attention network scores (in RT) and accuracy (%) of CD patients and HCs.

	CD (*N* = 29) Mean ± SE	HC (*N* = 26) Mean ± SE	*Z/t*	*P*
Alerting^a^	9.86 (27.95)	33.62 (23.41)	−3.40	0.01
Orienting^a^	33.03 (34.84)	30.65 (32.05)	0.26	0.79
Executive control^b^	115.45 (67.32)	113.73 (55.52)	−0.55	0.58
Total Mean RT^a^	702.17 (106.86)	709.38 (94.84)	−0.26	0.79
Accuracy^b^	96.24% (3.33%)	97.62 (2.02%)	−1.67	0.09

a Normally distributed, intergroup comparison using the independent-sample *t*-test.

b Nonnormally distributed data, intergroup comparison using the Mann–Whitney *U* test.

### Correlations

The Spearman correlation test revealed that the efficiency of the alerting network in the two groups was negatively correlated with age (*r_s_* = −0.561, *p* = 0.002; *r_s_* = −0.622, *p* = 0.001) and positively correlated with education level (*r_s_* = 0.384, *p* = 0.040; *r_s_* = 0.478, *p* = 0.014) and MoCA-BJ scores (*r_s_* = 0.499, *p* = 0.006; *r_s_* = 0.395, *p* = 0.046) but had no significant interaction with HAMA and HAMD scores. There was no significant interaction between the scores of the orienting network and executive control network and age, education level, MoCA-BJ, HAMA, or HAMD scores in the two groups. The efficiency of the alerting network in the CD group was significantly negatively correlated with the TWSTRS torticollis severity scale score and disability scale score (*r_s_* = −0.824, *p* < 0.001 and *r_s_* = −0.75, *p* < 0.001, respectively) and was moderately negatively correlated with the TWSTRS pain scale score (*r_s_* = −0.428, *p* = 0.021). The CD group orienting network efficiency was moderately negatively correlated with the TWSTRS torticollis severity scale score and disability scale score (*r_s_* = −0.569, *p* = 0.001 and *r_s_* = −0.81, *p* = 0.001, respectively) and had no significant interaction with the TWSTRS pain scale score (*r_s_* = −0.169, *p* = 0.380). The executive control network efficiency in the CD group was weakly negatively correlated with the TWSTRS torticollis severity scale score (*r_s_* = −0.388, *p* = 0.037) and had no significant interaction with the TWSTRS disability scale score or the pain scale score (*r_s_* = −0.324, *p* = 0.87 and *r_s_* = −0.188, *p* = 0.328, respectively). In addition, the total mean RT of CD patients was not correlated with the scores on any subscale of the TWSTRS. Accuracy in CD patients was negatively correlated with TWSTRS torticollis severity and disability (*r_s_* = −0.417, *p* = 0.025; *r_s_* = −0.376, *p* = 0.044) but not with pain scale scores.

## Discussion

In this study, we applied the ANT to CD patients to investigate attentional networks. A lower alerting effect was observed, whereas no differences in the orienting or executive control networks were shown in CD patients compared with HCs. In addition, there was no difference in the total mean RT or accuracy between the two groups. The alerting network function in both groups was negatively correlated with age and positively correlated with educational attainment and MOCA-BJ. We also found that the three attentional network functions were negatively correlated with the severity of CD, with the strongest correlations in alerting network function.

The inefficiency of the alerting network was observed in CD patients, which indicates that the difference in RT between the two types of test conditions without a warning cue and with a double cue is small and that CD patients do not benefit from time warnings as HCs do. In the tests with no cue congruence, the CD patients had a shorter mean RT than the HCs (718.14 ms vs. 734.04 ms); however, in the tests with double cue congruence, the CD patients had a longer mean RT than the HCs (696.59 ms vs. 692.85 ms), indicating that CD patients lack the ability to use additional useful information from warning cues to improve their speed of response. This finding forecasts that the use of a warning cue to speed up the RT of CD patients may fail.

Alerting refers to maintaining the body in a responsive state to receive information. Previous studies of brain regions involved in alertness control through neuroimaging have shown that the thalamus and frontal and parietal regions, especially in the right hemisphere, are activated (Fan et al., 2002, 2005). Several studies have shown that CD patients have abnormalities in brain regions such as the thalamus, prefrontal lobe, and right medial superior frontal gyrus (Li et al., 2017; Groth et al., 2020; Wei et al., 2021). This finding is consistent with the results of the present study. A series of animal and clinical studies have confirmed that alertness is closely related to the norepinephrine transmitter in the brain (Aston-Jones et al., 1994; Witte and Marrocco, 1997; Coull et al., 2001; Beane and Marrocco, 2004). However, no studies have reported on the levels of norepinephrine in the brains of CD patients. From the results of our study, it is speculated that CD patients may have a reduced level of norepinephrine in the brain; however, further research is needed to confirm this hypothesis.

The function of orienting networks is to selectively process incoming information. Our results indicate that there was no statistically significant difference between the two groups in orienting network efficiency. This result indicated that CD patients had the same sensitivity to spatial cues as normal controls, and their orienting network function was intact. This finding is not specific to CD patients. Previous studies have revealed that patients with Wilson disease and essential tremor also have intact orienting network function (Han et al., 2014; Pauletti et al., 2015). Furthermore, it has been demonstrated that the cholinergic system emerging in the basal forebrain plays a key role in the orienting network (Petersen and Posner, 2012). A recent study demonstrated functional cholinergic deficits in CD patients with pedunculopontine nucleus choline acetyltransferase deficiency (Mente et al., 2018). However, they also found no difference in the numbers of putaminal cholinergic neurons in CD patients compared with controls. The results of the present study do not support the existence of cholinergic system abnormalities in CD patients, and further research is needed to confirm this hypothesis.

The function of the executive control network is to make plans and monitor and resolve conflicts. This study showed that there was no statistically significant difference in the efficiency of the executive control network between the two groups. This result is consistent with the findings of Burke et al., who reported that CD patients had no observed deficits in multimodal measures of executive function (Burke et al., 2020).

There was no significant global delay in total mean RT in CD patients. Total mean RT did not appear to be related to disease severity, implying that impaired presentation may be the primary manifestation of disease rather than symptoms secondary to torticollis. These data are consistent with those published by Willimas et al., who reported no reduction in response time to luminophore stimulation in CD patients (Williams et al., 2020). This study revealed that the level of accuracy in CD patients was affected by the severity of torticollis and disability. However, the CD patients showed a similar level of accuracy as HCs, which may be related to the lower TWSTRS scores of the patients entering our study.

Through correlation analysis, we showed that the alerting, orienting, and executive control network functions of CD patients were all affected by the severity of torticollis. The greater the severity of CD, the worse the scores on the three attentional network functions. However, the alerting network efficiency of CD patients was significantly lower than that of HCs, but the orienting and executive control network efficiency were similar between the two groups. These results suggest that the impaired alerting network in CD patients cannot be explain solely by factors such as abnormal neck posture, which may be involved in the pathophysiological mechanisms of the disease. Undeniably, the effect of torticollis on the patient’s attention function is objective. Further research is needed to explore whether attention deficits are reversed when patients return to normal after receiving botulinum toxin A injections. We also found that the alerting network function of both groups was negatively correlated with age and positively correlated with years of education and intelligence level. Our results are in accordance with previous research conclusions (Gamboz et al., 2010; Cromarty et al., 2018). In addition, a study has shown that chronic pain can lead to impaired attention function (Yoshino et al., 2021), which was confirmed in our study. We found that TWSTRS pain scale scores in CD patients were inversely correlated with alerting network function but not with orienting and executive control network function. Therefore, chronic pain may be one of the factors that contributes to impaired alerting network function in CD patients, despite the low pain scores of the enrolled patients. Studies have revealed that anxiety and depression can lead to impaired attention function (Pacheco-Unguetti et al., 2011; Schock et al., 2011), which is inconsistent with our findings. Our results suggest that the attentional network function of CD patients has no correlation with HAMA and HAMD scores, which may be due to the low HAMA and HAMD scores of the enrolled patients.

This study revealed that CD patients have defects in the attention network, especially the low efficiency of the alerting network, which is an interesting discovery. This discovery has important value for the follow-up research and treatment of CD patients. Research has shown that attention function training can improve the efficiency of attention networks, as well as language and motor function. Liu et al. (2019) used fingertip-based adaptive force control tasks to train attention function and found that the efficiency of the executive control network during ANT was significantly improved after training. Zhang et al. (2019) found that gradual attention training seems to improve the language function of poststroke aphasia patients. Allcock et al. (2009) studied the effects of computer-programed attention tasks in Parkinson’s patients and found that attention function training can improve patients’ walking ability and reduce the frequency of falls. Therefore, attention function training may become a new direction for the treatment of patients with CD. In the future, we can further study the effect of attention function training on the movement and cognitive function of patients with CD.

## Conclusion

In summary, the attention network was impaired in patients with CD, which was mainly specific to the alerting network but not the orienting or executive control network. In addition, the alerting, orienting, and executive control network functions of CD patients were all affected by the severity of torticollis, especially the alerting network function.

## Limitations

Due to the low incidence rate of CD, this study failed to include a sufficient sample size, resulting in an obviously uneven number of cases among different subtypes. We failed to compare the attention network function of patients with different subtypes of CD. In future studies, we will need to include enough cases to compare the differences in attention network function among patients with different CD subtypes. In addition, some of the CD patients enrolled in this study failed to undergo genetic testing.

## Data availability statement

The raw data supporting the conclusions of this article will be made available by the authors, without undue reservation.

## Ethics statement

The studies involving human participants were reviewed and approved by Affiliated Hospital of Institute of Neurology of Anhui University of traditional Chinese medicine. The patients/participants provided their written informed consent to participate in this study.

## Author contributions

KX and YH conceived and designed the experiments. KX, LZ, SS, and RR performed the experiments. KX, SS, and LH analyzed the data. KX and SH contributed to reagents, materials, and analysis tools and wrote the paper. All authors contributed to the article and approved the submitted version.

## Conflict of interest

The authors declare that the research was conducted in the absence of any commercial or financial relationships that could be construed as a potential conflict of interest.

## Publisher’s note

All claims expressed in this article are solely those of the authors and do not necessarily represent those of their affiliated organizations, or those of the publisher, the editors and the reviewers. Any product that may be evaluated in this article, or claim that may be made by its manufacturer, is not guaranteed or endorsed by the publisher.
